# Wisconsin State Board of Dental Examiners

**Published:** 1900-01

**Authors:** 


### Domestic Correspondence.

### WISCONSIN STATE BOARD OF DENTAL EXAMINERS.

To the Editor:

Sir,—It is well known to the members of the dental profession, especially those interested in dental education, that in April, 1899, the Wisconsin State Board of Dental Examiners refused to register diplomas from the Chicago dental colleges and other schools, as the law provides. The provision of the law is that the Board shall at all times issue a license to any regular graduate of any reputable, legally incorporated dental college, without examination, upon the payment of the registration fee. After making inquiry of the secretary of the Board as to the reason why the diploma of his client was not registered, Attorney Quarles, who had been retained in the case, received the following reply:

"MILWAUKEE, April 15, 1899.

"HON. J. V. QUARLES, Milwaukee, Wis.:

"DEAR SIR,—I am authorized to say, from instructions received from a member of the Committee on Colleges of the National Association of Dental Examiners that if the college you represent accepts all the rules as laid down by the National Association of Dental Examiners, in regular form through that body, that this Board will, upon the receipt of such knowledge, issue licenses to regular graduates of said College.

(Signed) "W. H. CARSON,
"*Secretary.*"

After receiving the above letter, Dr. P. T. Diamond, a graduate of the Chicago College of Dental Surgery brought mandamus proceedings to compel the board to accept his diploma. The Board moved to quash the proceedings, which motion was denied by the court in a vigorous decision handed down by Judge Sutherland, of the Superior Court of Milwaukee County, Wis. Summing up the case, in regard to the standing of the college, the Judge makes use of the following language:

"The relation in this case shows that among intelligent men, whether members of the dental profession or not, the Chicago College of Dental Surgery must be regarded as a reputable institution. . . . Therefore, without difficulty, the Court reaches the conclusion that the motion to quash the mandamus proceedings must be denied."

The action of the Board was based on the ground that those schools refused to subscribe to a rule passed by the National Association of Dental Examiners, regarding the preliminary educational qualification of students.

The National Association of Dental Examiners, of which the Wisconsin Board was a member, at their meeting at Niagara Falls, in August, 1899, rescinded the rule which was the cause of the controversy, and passed a resolution adopting, in substance, the rule governing preliminary educational qualifications of students which was adopted in 1898 by the National Association of Dental Faculties. In adopting this resolution the National Association of Dental Examiners recommended to the various State Boards that all the schools belonging to the National Association of Dental Faculties be placed on the recognized list, and that the graduates of those schools be licensed, and that all litigation cease. In all States where difficulties had arisen regarding the registration of diplomas of graduates of schools belonging to the National Association of Dental Faculties, the trouble was at once terminated and licenses issued, except in the State of Wisconsin. The representative from the Wisconsin Board pledged himself at Niagara Falls to return home and do all in his power to terminate the litigation. The week following the National Association meeting, the Wisconsin Board, with their attorney, met by appointment the representatives of the Chicago College of Dental Surgery and the plaintiff in the case against the Board with his attorney, and after a conference the representatives of the Board informed the representatives of the college that the members of the Board had voted *unanimously* to continue the litigation.

On August 13, 1899, the following letter was written by Senator J. V. Quarles, attorney for the complainant, to Dr. T. W. Brophy, Dean of the Chicago College of Dental Surgery:

"Quarles, Spence & Quarles,
Attorneys and Counsellors,
The Sentinel Building.

"Milwaukee, Wis., August 13, 1899.

"Dr. T. W. Brophy, No. 126 State Street, Chicago, Ill.:

"Dear Doctor,—As you are aware, a meeting of the State Board of Dental Examiners took place yesterday in this city for the ostensible purpose of carrying out the recommendations of the National Board so explicitly made at its meeting at Niagara Falls. Nothing could be more plain and explicit than the recommendations of such National Association, which ought to be looked upon as a command by members thereof.

"I have to report, however, that our State Board have assumed to be wiser than the national organization, and have positively declined to follow or respect the mandate of the central body. The State Board refuses to recognize the diplomas of your college and all others similarly situated, and leaves no course open but to continue the litigation. We shall, therefore, unless ordered to the contrary, embrace the first opportunity to crowd the case to a final hearing and allow the National Board to deal with its recalcitrant members.

"Very respectfully yours,
(Signed) "Quarles, Spence & Quarles."

Preparations were then made for a vigorous prosecution of the case. The Committee on Law of the National Association of Dental Faculties, which was created at the Niagara meeting in August, 1899, for the purpose of taking charge of this litigation, as well as any other litigation involving the Association or any college holding membership therein, held a meeting in Chicago, October 14, 1899, and after a conference with the members of the Wisconsin State Board, the latter agreed to license graduates of the Chicago colleges and all schools belonging to the National Association of Dental Faculties. November 6 the agreement was consummated. November 7 the following letter was received by the Dean of the Chicago College of Dental Surgery:

"QUARLES, SPENCE & QUARLES,
Attorneys and Counsellors,
The Sentinel Building.

"MILWAUKEE, WIS., November 7, 1899.

"DR. T. W. BROPHY, Chicago, Ill.:

"DEAR DOCTOR,—After great tribulation, regarding matters of detail, I am glad to report to you that the Board has finally decided to conform with the provisions of the dental law of Wisconsin, abide by the ruling of the National Association of Dental Examiners, and license Chicago graduates and all other graduates from schools holding membership in the National Association of Dental Faculties, thus admitting that in their action in refusing to license these graduates from April 11 to November 6, 1899, they were in the wrong. Everything, consequently, in the Diamond mandamus case has been brought to a satisfactory conclusion.

"The injustice the Wisconsin State Board of Dental Examiners has done your graduates, yourself, and the many schools involved cannot be easily forgotten, but our success in securing all we contended for is an assurance of the justice of our cause.

"In evidence of our victory, Dr. Diamond's license has been issued, the stipulation to discontinue the case has been signed by both parties, the whole matter is closed up, and the litigation is a thing of the past.

"Yours truly,
"QUARLES, SPENCE & QUARLES."

A. O. HUNT,
W. C. BARRETT,
HENRY W. MORGAN,
*Committee on Law of the National Association of Dental Faculties.*

November 17, 1899.

